# KSHV miRNAs Decrease Expression of Lytic Genes in Latently Infected PEL and Endothelial Cells by Targeting Host Transcription Factors

**DOI:** 10.3390/v6104005

**Published:** 2014-10-23

**Authors:** Karlie Plaisance-Bonstaff, Hong Seok Choi, Tyler Beals, Brian J. Krueger, Isaac W. Boss, Lauren A. Gay, Irina Haecker, Jianhong Hu, Rolf Renne

**Affiliations:** 1 Department of Molecular Genetics and Microbiology, University of Florida, Gainesville, FL 32610, USA; E-Mails: karliebonstaff@gmail.com (K.P.-B.); hschoi@ufl.edu (H.S.C.); tbeals@ufl.edu (T.B.); brian.krueger@duke.edu (B.J.K.); iboss2022@gmail.com (I.W.B.); lagay@ufl.edu (L.A.G.); irina25@me.com (I.H.); Jianhong.Hu@bcm.edu (J.H.); 2 UF Health Cancer Center, University of Florida, Gainesville, FL 32610, USA; 3 UF Institute of Genetics, University of Florida, Gainesville, FL 32610, USA

**Keywords:** KSHV, miRNA, lytic reactivation, latency

## Abstract

Kaposi’s sarcoma-associated herpesvirus (KSHV) microRNAs are encoded in the latency-associated region. Knockdown of KSHV miR-K12-3 and miR-K12-11 increased expression of lytic genes in BC-3 cells, and increased virus production from latently infected BCBL-1 cells. Furthermore, iSLK cells infected with miR-K12-3 and miR-K12-11 deletion mutant viruses displayed increased spontaneous reactivation and were more sensitive to inducers of reactivation than cells infected with wild type KSHV. Predicted binding sites for miR-K12-3 and miR-K12-11 were found in the 3’UTRs of the cellular transcription factors MYB, Ets-1, and C/EBPα, which activate RTA, the KSHV replication and transcription activator. Targeting of MYB by miR-K12-11 was confirmed by cloning the MYB 3’UTR downstream from the luciferase reporter. Knockdown of miR‑K12-11 resulted in increased levels of MYB transcript, and knockdown of miR-K12-3 increased both C/EBPα and Ets-1 transcripts. Thus, miR-K12-11 and miR-K12-3 contribute to maintenance of latency by decreasing RTA expression indirectly, presumably via down‑regulation of MYB, C/EBPα and Ets-1, and possibly other host transcription factors.

## 1. Introduction

Kaposi’s sarcoma-associated herpesvirus (KSHV), also known as Human Herpesvirus 8 (HHV-8), is a DNA tumor virus that infects endothelial cells *in vivo* and is the etiological agent of Kaposi’s sarcoma (KS) [1]. KSHV has been linked to two B cell lymphoproliferative disorders, primary effusion lymphoma (PEL) and a subset of multicentric Castleman’s disease (MCD) [2,3]. As with all herpesviruses, KSHV has both lytic and latent modes of replication. During lytic replication and reactivation, genome-wide expression occurs in a temporally regulated cascade of immediate early, early, and late genes, which results in lysis of the host cell and release of progeny virus. During latency most tumor cells express only a limited number of genes, the majority residing in the latency-associated region, which encodes the latency-associated nuclear antigen (LANA), v-FLIP, v-cyclin, kaposin, and 12 miRNA genes. A subset of cells also express vIRF3, vIL-6, and K1 during latency [4,5,6,7,8,9,10,11,12].

MicroRNAs (miRNAs) are non-coding RNAs 19–23 nucleotides in length that regulate gene expression post-transcriptionally by targeting 3' untranslated regions (UTRs) of messenger RNAs (for review see [13]). Since the discovery of highly expressed KSHV-encoded miRNAs in all KSHV-associated tumors [14,15], several cellular targets of KSHV-encoded miRNAs have been identified. Roles for KSHV miRNAs include promotion of angiogenesis, cell cycle regulation, inhibition of apoptosis and recently transformation [16] (for reviews see [17,18,19]). Early after the discovery of herpesvirus-encoded miRNAs it was hypothesized that these novel viral post‑transcriptional regulators may promote latency by targeting lytic genes [20]. Indeed, one EBV microRNA, miR-BART2, is encoded antisense to BALF5, the EBV DNA polymerase, and targeting and cleavage of the BALF5 mRNA has been experimentally confirmed [21,22]. For KSHV, elegant work from the Ganem lab utilizing miRNA mimic- and antagomir-based screens provided evidence that KSHV miRNAs can modulate the latent/lytic transition through direct targeting of RTA by miR-K12-9 * [23]. RTA, the product of the immediate early lytic gene ORF50, is the KSHV replication and transcriptional activator that is expressed first during reactivation and initiates the cascade of lytic gene expression by activating several early lytic gene promoters [24,25]. Direct miRNA targeting of RTA was also demonstrated for miR-K12-5 and miR-K12-7 based on *in vitro* luciferase assays [26,27], consistent with *in silico* prediction of targets within the RTA 3'UTR. Hence, direct targeting of RTA by miRNAs may act as a gate keeper of latency by preventing reactivation.

Conceptually, miRNAs can also contribute to latency by targeting host factors that normally tip the balance toward reactivation. We present evidence here that KSHV miRNAs contribute to the maintenance of latency by targeting cellular transcription factors. Our data demonstrate that miR-K12-11 and miR-K12-3 prevent lytic reactivation by modulating expression of the transcription factors MYB, C/EBPα and Ets-1, which have previously been reported as activators of the RTA promoter [28,29,30].

## 2. Materials and Methods

### 2.1. Cell Lines

BC-3-G cells containing a PAN-GFP expression cassette were provided by Ren Sun (UCLA) [28]. BC-3-G, BC-3 and BCBL-1 primary effusion lymphoma (PEL) cells are latently infected with KSHV, and were cultured in RMPI supplemented with 10% FBS, 1% penicillin/streptomycin, and 1% sodium pyruvate. Human embryonic kidney 293 and 293T cells were cultured in DMEM with 10% FBS and 1% P/S. iSLK cells were kindly provided by Don Ganem (UCSF) [31] and were cultured under the same conditions as 293 cells. TIVE (Telomerase Immortalized Vein Endothelial) cells were cultured as previously described [32].

### 2.2. Antagomir Derepression Assays and Quantitative Reverse Transcription-PCR (RT-qPCR) Analysis

For inhibition of KSHV miRNAs, 2'OMe RNA antagomirs were used as previously described [33]. PEL cells (1 × 10^6^) were transfected with 50–400 nM of antagomir using TransIT-TKO transfection reagent (Mirus) as described [34]. At 48 h post transfection (hpt), cells were harvested using RNA-Bee (Tel-Test) according to the manufacturer’s instructions. 1 μg of DNase treated RNA was reverse transcribed using SuperScript III (Invitrogen, Grand Island, NY, USA) according to the manufacturer’s instructions. Quantitative PCR (qPCR) analysis was carried out using an ABI StepOne Plus system along with ABI Fast SYBR reagent (Applied Biosystems, Carlsbad, CA, USA). Expression of all genes was normalized to β-actin expression, and Student’s t-tests were performed to determine statistical significance compared to the mock control. The sequences of PCR primers can be found in Table 1.

**Table 1:** Primers used in this study.

Gene	Primer Sequence 5'-3'	Reference
MYB 3'UTR	FWD- GATGGAGGAGCAGATGACATC REV- AGGTAAAATAAGGGCAC	
Ets-1 3'UTR	FWD- CGTGTTGGTTGGACTCTGAA REV- TCTCCAGCAAAATGATGTGC	
C/EBPα 3'UTR	FWD-CTTGTGCCTTGGAAATGCAAACTCACC REV- AAGAAGAGAACCAAGCCGTCCTTC	
MYB	FWD- TCAGGAAACTTCTTCTGCTCACA REV- AGGTTCCCAGGTACTGCT	
Ets-1	FWD- AAGGGAGATCGAAGGAGGAA REV- TCTGCTATAGGAACTGCAGGAG	
C/EBPα	FWD- TGTATACCCCTGGTGGGAGA REV- TCATAACTCCGGTCCCTCTG	
RTA	FWD- CACAAAAATGGCGCAAGATGA REV- TGGTAGAGTTGGGCCTTCAGTT	[35]
ORF59	FWD- TTAGAAGTGGAAGGTGTGCC REV- TCCTGGAGTCCGGTATAGAATC	[36]
ORF19	FWD- GGCGAAAAAGTCAGCGGTGGT REV- CGGCGCGTCTTCCCTAAAGA	[37]
LANA N-Terminus	FWD- GCGCCCTTAACGAGAGGAAGTT REV- TTCCTTCGCGGTTGTAGATG	
β-actin	FWD- CATGTACGTTGCTATCCAGGC REV- CTCCTTAATGTCACGCACGAT	Primerbank ID 4501885a1

### 2.3. Virus Isolation and Quantitation

Virus particles were harvested from PEL cells 6 days post transfection (dpt) with antagomirs. Cells were pelleted at 1100 RPM for 5 min and media supernatant was passed through a 0.45 μM filter. Virus particles were pelleted by ultra-centrifugation using a Beckman SW-40 rotor at 100,000 g for 1 h on a 25% sucrose cushion. Virus pellets were resuspended in 1% of the original medium volume using serum-free RPMI. DNA was extracted from 25 μL of virus stocks using DNAzol (Molecular Research Center, Inc., Cincinnati, OH, USA) and resuspended in 25 μL of ddH_2_O. Viral genome copy number was determined by qPCR assay using a standard curve based on serially diluted LANA expression plasmid.

### 2.4. Recombineering of miRNA Deletion Mutants in KSHV BAC16

KSHV BAC16 [38,39] was kindly provided by the Jung lab (USC). A modified version of the protocol by Tischer *et al.* [40] was used to generate mutant bacmids. BAC DNA was isolated from bacteria using the Large-Construct Kit (Qiagen) according to the manufacturer’s recommendations. 293T cells were transfected with 2 μg of DNA using TransIT-293 reagent (Mirus) according to the manufacturer’s instructions. Cells were selected with 100 μg/mL hygromycin B and expanded for 10–15 days. Transfected cells, which express GFP, were monitored under a fluorescence microscope. Once the expanded cell population was 100% GFP positive, cells were induced with 20 pg/mL of TPA and 1 mM valproic acid and co-cultured with iSLK cells [41]. Infected iSLK cells were selected using 1 μg/mL puromycin, 250 μg/mL G418, and 1.2 mg/mL hygromycin B. Stable KSHV∆miRNA BAC16 iSLK cells were induced using 1 μg/mL doxycycline (DOX) and 1 mM sodium butyrate. Virus was collected and quantified at 4 days as described above.

### 2.5. Reporter Construction and Luciferase Assays

MYB, C/EBPα, and Ets-1 full length 3'UTR sequences were cloned downstream of the luciferase gene in pGL3 promoter (Promega) using pCRII-TOPO (Invitrogen) for MYB and GeneArt Seamless Cloning (Invitrogen) for C/EBPα and Ets-1. Primers for 3'UTR cloning can be found in Table 1. 293 cells were transfected using TransIT-293 reagent (Mirus) in 24-well cell culture dishes according to the manufacturer’s protocol. Each transfection reaction contained 2 ng pCMV-Renilla (Promega) control vector, 20 ng pGL3 promoter-3'UTR reporter construct and 0, 400 or 800 ng pcDNA3.1 miRNA expression vector complemented with 800, 400, or 0 ng of empty pcDNA3.1 vector as filler to reach 800 ng total pcDNA3.1 in each transfection [42]. Cells were harvested 72 h post transfection and luciferase activity was quantified using the Dual Luciferase Reporter kit (Promega) according to the manufacturer’s protocol. Each lysate was assayed for firefly luciferase activity using a FLUOstar OPTIMA reader (BMG Labtech, Cary, NC, USA). Firefly luciferase activity for each sample was normalized to Renilla expression and samples were compared to the miRNA mock transfection control. Transfection assays were performed in triplicate and repeated at least 3 times. Standard deviation was calculated for triplicates and displayed as error bars in the figures. Significance of the repression of the reporter construct relative to the 0 ng miRNA expression vector was tested by one-tailed, unpaired t-test.

## 3. Results

### 3.1. Screening for Effects of KSHV miRNA Knockdown on Reactivation in BC-3-G Cells

In order to determine which KSHV-encoded miRNAs may affect lytic reactivation, miRNA knockdown studies were performed in BC-3-G [28], a PEL-derived indicator cell line. BC-3-G cells contain a GFP cassette under the control of the KSHV PAN promoter, which is highly transactivated by RTA. Upon lytic reactivation, RTA is the first protein expressed, activates the PAN promoter and as a result switches on GFP expression [28].

**Figure 1:**
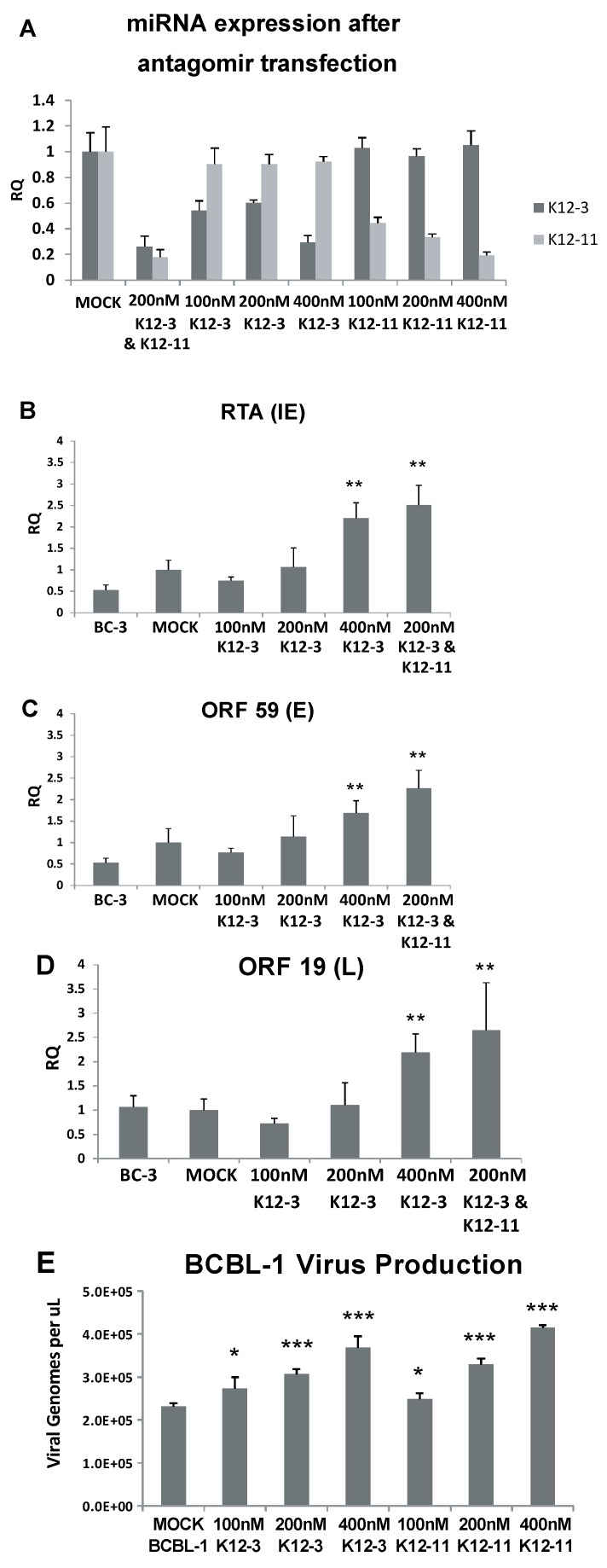
Lytic gene expression and virus production following miR-K12-3 and miR-K12-11 knockdown in PEL cells. (**A**) Confirmation of miRNA knockdown after antagomir transfection. BCBL-1 cells were transfected with the indicated amounts of miR-K12-3 or miR‑K12-11 antagomir or a combination of both. RNA was harvested at 48 h after transfection and TaqMan miRNA-RT and qPCR was performed using primers and probes specific to miR-K12-3 and miR-K12-11. Primers and a probe specific to RNU66 were used as a loading control and all samples were normalized to mock transfected controls; (**B**–**D**) BC-3 cells were transfected with the indicated amounts of miRNA-specific antagomir. Total RNA was collected at 48 h post-transfection and reverse transcribed; qPCR was performed using primers specific for RTA (**B**); ORF59 (**C**); and ORF19 (**D**); All samples were normalized to β-actin expression and compared to gene expression in the mock transfected control; (**E**) BCBL-1 cells were transfected with the indicated amounts of miRNA-specific antagomir. Cell media harboring progeny virus was collected at 6 days post-transfection, and the genome DNA concentration determined by qPCR using plasmid DNA as standard. *p* < 0.05 (*), *p* < 0.01 (**), *p* < 0.001 (***) compared to gene expression in the mock transfected control.

To validate efficiency and specificity of 2'OMe antagomirs against KSHV miRNAs, knock-down was assessed by stem-loop TaqMan qPCR which measures the amount of mature miRNA. These experiments were done in BCBL-1 cells with antagomirs against miR-K12-3 and miR-K12-11. Transfection of antagomirs against miR-K12-3 yielded reduced levels of miR-K12-3, with the greatest effect at the highest concentration (400 nM) of antagomir (Figure 1A). Antagomirs against miR-K12-3 did not reduce the level of miR-K12-11. Similarly, miR-K12-11 antagomirs gave progressively reduced levels of miR-K12-11 with increasing antagomir concentration, but had no effect on the level of mature miR-K12-3 (Figure 1A). When antagomirs against miR-K12-3 and miR-K12-11 were co‑transfected, strong inhibition of both miRs was observed.

BC-3-G cells were transfected with antagomirs for each of the 12 KSHV-encoded miRNAs. Cells were monitored for GFP expression by fluorescence microscopy at 72 h after transfection. Mock transfected control cells displayed a small number of GFP-expressing cells arising from spontaneous reactivation. Screening was performed with inhibition of all 12 KSHV-encoded miRNAs individually or in combinations. The greatest increase in GFP expression was observed when either miR-K12-3 or miR-K12-3 in combination with miR-K12-11 were knocked down (data not shown). MiR-K12-3 is the most abundant miRNA within RISC complexes in BC-3 cells but is only moderately expressed in BCBL-1 cells; miRK-12-11 is moderately expressed in both PEL lines. [43,44]. Interestingly, miR-K12-11 is a mimic of hsa-miR-155 and has been shown to play an important role in B cell proliferation [33,34,35,36,37,38,39,40,41,42,43,44,45]. To take into account miRNA expression differences between PEL lines, we tested the effects of antagomirs against miR K12-3 and miR-K12-11 in both BCBL-1 and BC-3 cells.

### 3.2. Lytic Gene Expression and Virus Production Increase upon miR-K12-3 and miR-K12-11 Knockdown in PEL Cells

Based on the preliminary results indicating that knockdown of miR-K12-3 and miR-K12-11 in BC-3-G cells increased RTA expression, we tested for effects on virus lytic gene expression downstream from RTA and on virus production. BC-3 cells, the parent of BC-3-G cells, were transfected with miR‑K12-3 antagomir, or a combination of miR-K12-3 and miR-K12-11. RT-qPCR was performed at 48 h to monitor expression of RTA (immediate early gene), ORF59 (early) and ORF19 (late glycoprotein). Significant increases in the levels of RTA, ORF59 and ORF19 transcripts were observed upon addition of 400 nM antagomir against miR-K12-3 (Figure 1B–D). Moreover, expression of these lytic genes was induced more efficiently when both miR-K12-3 and miR-K12-11 were knocked down simultaneously (200 nM miR-K12-3 antagomir plus 200 nM miR-K12-11 antagomir (Figure 1B–D).

We asked if the increase in lytic gene expression translates into production of progeny virus. Latently infected BCBL-1 cells were used for these experiments. Cell-free virus was isolated from BCBL-1 supernatants 6 days post antagomir transfection, viral DNA was extracted, and viral genome copy number determined by qPCR. Figure 1E shows that virus production increased in a dose dependent manner with 100, 200 and 400 nM antagomir when either miR-K12-3 or miR-K12-11 was knocked down. These results indicate that knockdown of miR-K12-3 alone or of miR-K12-3 and miR-K12-11 together increases expression of lytic genes belonging to the immediate early, early and late classes. Knockdown of miR-K12-3 or of miR-K12-11 alone was sufficient to increase production of progeny virus.

### 3.3. Generation of KSHV miRNA Deletion Mutants and Latently Infected iSLK Cells

In order to investigate the role of miR-K12-3 and miR-K12-11 in the context of viral infection, we generated two miRNA deletion mutants using the KSHV bacmid BAC16, which was derived from the PEL cell line JSC-1 [38,39]. 20 to 25 bp regions were deleted from one arm of each pre-miRNA, destroying pre-miRNA hairpin formation without affecting neighboring miRNA expression. A modified version of the protocol of Tischer *et al.* was used to create markerless microRNA deletions within BAC16 [40,46]. Recombinant bacmids were validated by PCR and pulsed field gel electrophoresis to monitor for intact terminal repeats. Detailed experimental procedures to recover recombinant bacmids in iSLK cells will be reported elsewhere [47]. miRNA deletion mutant and wild-type (WT) bacmids were transfected into 293T cells. After selection, BAC16-containing 293T cells were induced with TPA and valproic acid, and co-cultured with iSLK cells, which harbor a doxycycline-inducible RTA gene and produce high levels of progeny virions [31,41]. WT BAC16-, KSHV∆miRK-12-3-, or KSHV∆miRK-12-11-containing iSLK cells produce high titer progeny virus after doxycycline induction (up to 1.14 × 10^7^ genome copies/mL). As a final quality control, episomal DNAs isolated from latently infected iSLK cells containing miRNA deletion mutants or WT BAC16 were analyzed by Illumina-based genome-wide sequencing, which confirmed the presence of the appropriate deletion without detecting any mutations outside the miRNA region.

**Figure 2:**
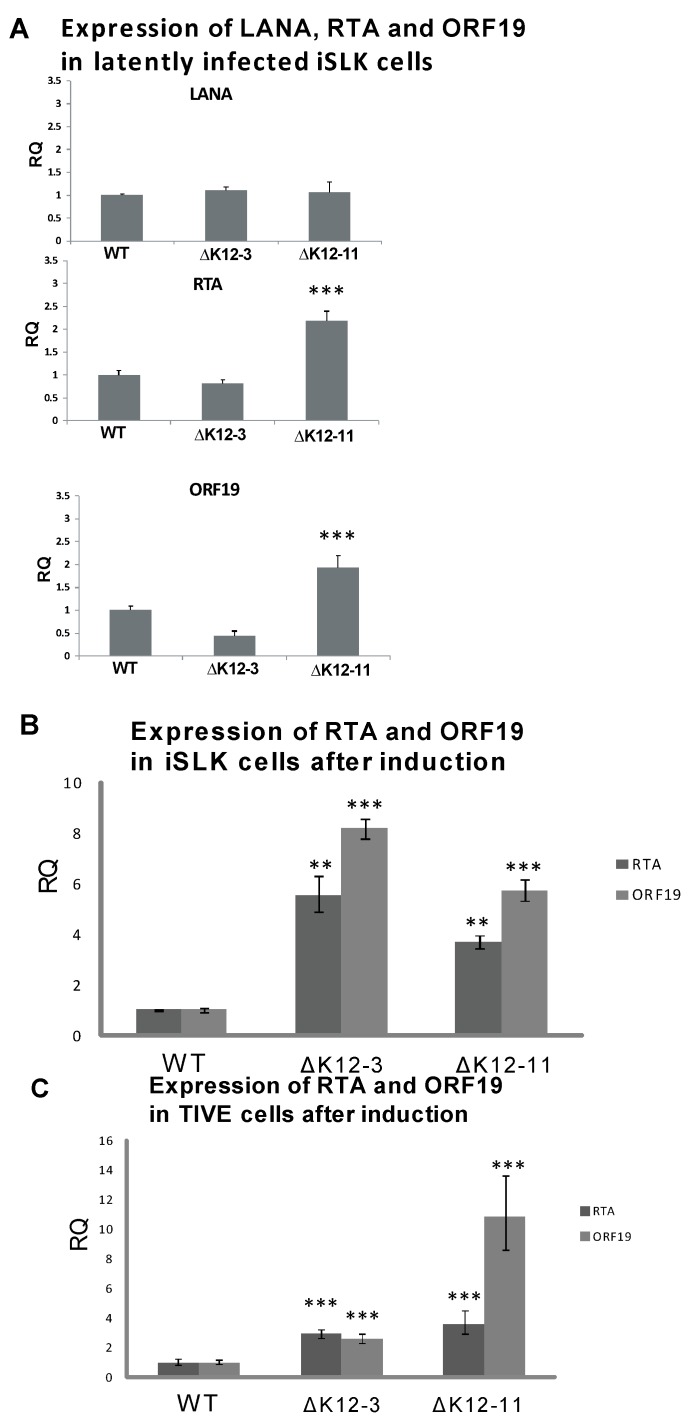
Expression of LANA and lytic genes in cells infected with KSHV miRNA deletion mutants. (**A**) iSLK cells latently infected with WT, ∆miR-K12-3, or ∆miR-K12-11 BAC16-derived KSHV were harvested for RNA and RT-qPCR was performed for LANA, RTA, and ORF19. All samples were normalized to β-actin expression; (**B**) Expression of RTA and ORF19 genes following induction of iSLK cells infected with WT, ∆miR-K12-3, or ∆miR-K12-11 KSHV mutants. Total RNA was harvested at 72 h post induction with 2 mM sodium butyrate and RT-qPCR performed for RTA and ORF19. All samples were normalized to β-actin and LANA expression. Samples were compared to the WT control. *p* < 0.05 (*), *p* < 0.01 (**), *p* < 0.001 (***); (**C**) Expression of RTA and ORF19 genes following induction of TIVE cells infected with WT, ∆miR-K12-3, or ∆miR-K12-11 KSHV mutants.

To test whether deletion of miR-K12-3 or miR-K12-11 had an effect on the maintenance of latency in iSLK cells, we analyzed spontaneous lytic gene expression during latent infection, and reactivation upon induction with sodium butyrate at a sub-optimal concentration. The expression levels of LANA determined by RT-qPCR in wild type- and mutant-infected iSLK cells were very similar (Figure 2A). However, iSLK cells latently infected with KSHV∆miR-K12-11 displayed higher expression levels of RTA and ORF19 than iSLK cells infected with either WT virus or KSHV∆miR-K12-3 (Figure 2A). Upon sub-optimal induction with 2 mM sodium butyrate, both KSHV∆miR-K12-3- and KSHV∆miR‑K12-11-infected iSLK cells displayed ≥4-fold higher levels of RTA expression compared to KSHV wild type infected iSLK cells, and ≥6-fold higher levels of ORF19 expression (Figure 2B). Recently it has been shown that SLK cells, which are commonly used for KS-derived endothelial tumor biology studies, are not of endothelial origin, but are of epithelial cell origin instead [48]. To test for lytic gene expression levels in endothelial cells infected with KSHV miRNA-deletion mutants, we repeated experiments in telomerase-immortalized vascular endothelial (TIVE) cells [32]. Sub-optimal induction of infected TIVE cells gave a significant increase in expression of RTA and ORF19 lytic genes on infection with either KSHV∆miR-K12-3 or KSHV∆miR-K12-11 compared with WT (Figure 2C), as was seen for iSLK cells (Figure 2B). These results suggest that expression of miR‑K12-3 and miR-K12-11 both contribute to the maintenance of latency in endothelial cells, and are consistent with the antagomir inhibition experiments in PEL cells which measured expression of RTA, ORF59, ORF19 and virus production during latent infection (Figure 1).

### 3.4. In Silico Seed Sequence Prediction for miR-K12-3 and miR-K12-11 Identified MYB, C/EBPα, and Ets-1 as Potential Targets

**Figure 3:**
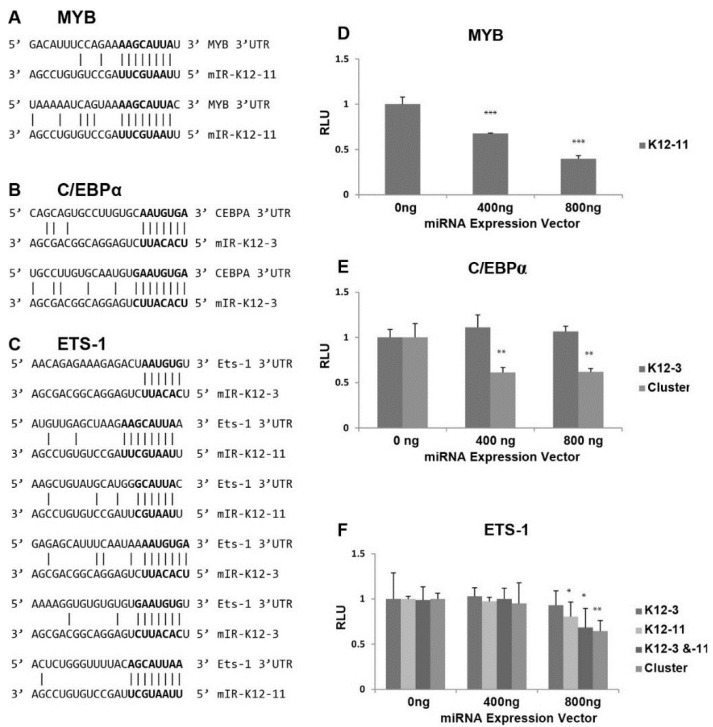
Effect of miRNAs on luciferase expression from 3'UTR reporter constructs. (**A**–**C**) Potential binding sites for miR-K12-3 and mIR-K12-11 in the 3'UTRs of the cellular MYB (**A**); C/EBPα (**B**) and Ets-1 (**C**) genes; (**D**–**F**) Dual luciferase reporter assays to assess targeting of the MYB (**D**); C/EBPα (**E**); and Ets-1 (**F**) 3'UTRs by KSHV miRNAs. 293 cells were transfected with pGL3-derived reporter plasmids carrying the MYB, C/EBPα or Ets-1 3'UTR downstream of the firefly luciferase together with the indicated amount of miRNA vectors expressing miR-K12-11 and/or miR-K12-3 or the 10 cluster miRNAs. Cells were harvested at 72 h post-transfection and the quantity of firefly luciferase measured by luminescence. Expression values were normalized to renilla luciferase expression from the pCMV-Renilla vector.

Scanning the 3'UTR of RTA revealed no seed sequence matches for miR-K12-3 or miR-K12-11. We therefore focused on transcription factors known to activate the viral RTA promoter [28,29,30], and searched for seed sequence matches of at least 6 nts [49]. The 3'UTR of the proto-oncogene MYB contained two potential 8 base binding sites for miR-K12-11 (Figure 3A). MYB is a transcriptional activator with a central role in hematopoiesis, and cooperates with transcription factors including members of the CEBP and Ets families. Targets of the MYB transcription factor include genes involved in development, cell survival, proliferation, and homeostasis [50]. C/EBPα is a member of the CCAAT/Enhancer-Binding Protein family, which are bZIP nuclear transcription factors. The 3'UTR of C/EBPα has two potential sites for miR-K12-3, each with a 7 base match (Figure 3B). C/EBPα is an important transcription factor involved in controlling tissue-specific gene expression in myeloid tissues and growth arrest [51].

Interestingly, we found 3 putative binding sites for miR-K12-3 and 3 for miR-K12-11 within the 3'UTR of Ets-1 (Figure 3C) ranging from 6 to 9 base matches. Ets-1 is a member of the Ets family of transcription factors, which are downstream effectors of the Ras-MAPK signaling cascades [52]. The Ets family binds to a specific DNA consensus sequence and activates genes involved in cell proliferation, differentiation, and survival [53].

The 3'UTRs of MYB, Ets-1, and C/EBPα were separately cloned downstream of the firefly luciferase reporter, and assays performed to test directly for miR-K12-3 and/or miR-K12-11 targeting of the resulting transcripts. The luciferase reporter plasmid DNAs were transfected with either a miR‑K12-3 or miR-K12-11 expression vector into 293 cells. Transfection with increasing amounts of miR-K12-11 expression vector gave progressively reduced luciferase activity from the luciferase‑MYB 3'UTR construct (Figure 3D), indicating that miR-K12-11 targets the MYB 3'UTR. Despite the presence of two predicted binding sites in the C/EBPα 3'UTR for miR-K12-3, miR-K12-3 expression alone did not cause significant reduction in luciferase expression from the C/EBPα 3'UTR construct (Figure 3E). The pcDNA3.1/cluster expression plasmid, which expresses 10 KSHV miRNAs [42], was also tested for effects on the C/EBPα 3'UTR, and significantly reduced luciferase expression (Figure 3E). Bioinformatic prediction revealed that the C/EBPα 3'UTR contains additional seed sequence matches for miR-K12-8 and -9 (data not shown). The 3.6 kb long 3'UTR of Ets-1 has a number of putative binding sites for KSHV miRNAs (miR-K12-1, -6, and -7) in addition to the sites for miR-K12-3 and miR-K12-11. The luciferase-Ets-1 3'UTR construct was cotransfected with plasmids expressing miR-K12-3 and miR-K12-11 alone and in combination, and with pcDNA3.1/cluster. Expression of miR-K12-3 alone had no significant effect, but miR-K12-11 alone and miR-K12-11 in combination with miR-K12-3 led to significant repression (Figure 3F). Expression of the 10 cluster miRNAs also gave reduced luciferase expression. These results suggest that Ets-1 is targeted by multiple KSHV miRNAs. In summary, these *in vitro* data show that all three transcription factors can be targeted by KSHV miRNAs.

### 3.5. MYB, C/EBPα and Ets-1 Expression Is Increased upon miRNA Knockdown in PEL Cells

**Figure 4:**
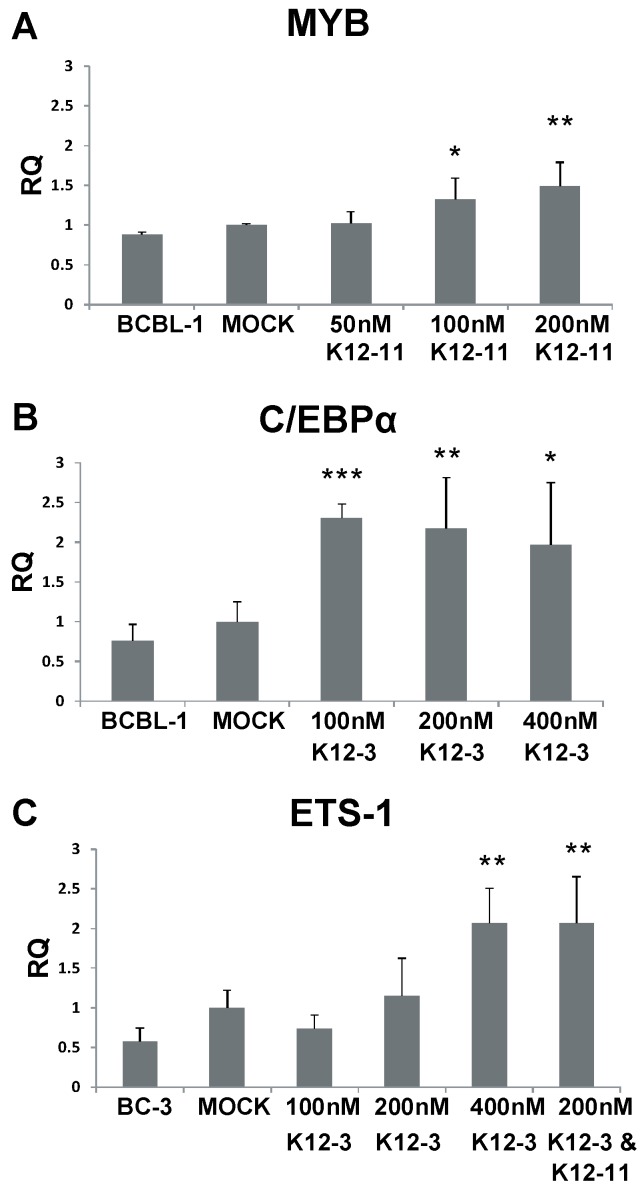
MYB, C/EBPα and Ets-1 expression after miR-K12-11 and miR-K12-3 knockdown in PEL cells. BCBL-1 and BC-3 cells were transfected with the indicated amounts of miRNA-specific antagomir. Total RNA was collected at 48 h, reverse transcribed, and MYB (**A**); C/EBPα (**B**); and Ets-1 (**C**) transcript levels determined by qPCR. All samples were normalized to β-actin expression and compared to gene expression in the mock transfected control. *p* < 0.05 (*), *p* < 0.01 (**), *p* < 0.001 (***).

To investigate whether MYB, C/EBPα, and Ets-1 are regulated by KSHV miRNAs in latently infected PEL cells, BCBL-1 and BC-3 cells were transfected with miR-K12-11 or miR-K12-3 antagomirs. RT-qPCR performed 48 h after antagomir transfection revealed increased levels of MYB transcript upon miR-K12-11 knockdown with dose dependence (Figure 4A). MYB transcript was increased 1.5-fold in BCBL-1 cells compared to mock-transfected control in the presence of 200 nM miR-K12-11 antagomir. For C/EBPα, transcript levels were increased approximately two-fold in BCBL-1 cells on addition of miR-K12-3 antagomir (Figure 4B). In BC-3 cells, where miR-K12-3 is the most highly expressed KSHV miRNA [44,54], knockdown of miR-K12-3 gave increases of up to 2-fold in Ets-1 transcript levels in a dose-dependent fashion (Figure 4C). Addition of 200 nM miR‑K12-3 and 200 nM miR-K12-11 antagomirs together gave a higher Ets-1 level than 200 nM miR‑K12-3 antagomir alone.

These data suggest that expression of KSHV miR-K12-11 in latent infection normally reduces the level of MYB transcript, and miR-K12-3 behaves similarly against C/EBPα and Ets-1. Our results point to a mechanism in which miR-K12-3 and miR-K12-11 down-regulate RTA expression by modulating cellular transcription factors in cells of both endothelial and lymphoid origin.

## 4. Discussion

We used BC-3-G cells containing GFP driven by the RTA-responsive PAN promoter to ask whether antagomir-based inhibition of KSHV miRNAs activates RTA. Inhibition of single or multiple miRNAs revealed that in this experimental system only antagomirs against miR-K12-3 and mir-K12-11 led to significantly increased RTA expression levels. We note that miR-K12-3 and miR-K12-11 expression levels are higher in BC-3 cells compared to BCBL-1 cells used in previous screens [43,44]. Antagomir inhibition of miR-K12-7 and miR-K12-5 did not activate RTA expression in BC-3-G cells, as was observed in 293 cells infected with Bac36-derived KSHV [26,27]. Additionally, miR-K12-9 * is not expressed at all in BC-3 cells due to the presence of a highly polymorphic miR-K12-9 pre-miRNA [55,56], and therefore RTA inhibition by miR-K12-9 * [57] would not be expected in this cell line. We note that recent ribonomics reports on KSHV miRNA targetomes using PAR-CLIP and HITS-CLIP [43,44] did not reveal direct targeting of RTA by KSHV miRNAs in two different PEL cell lines including BC-3 during latency. In contrast, targeting of host BCLAF1 by mIR-K12-5 has been reported to sensitize cells for reactivation [23], and BCLAF1 was validated as a viral miRNA target in BCBL-1 cells by PAR-CLIP and HITS-CLIP [43,44].

Since neither the 3'UTR of RTA nor its coding region contains any mir-K12-3 or miR-K12-11 seed sequence matches, mir-K12-3 and miR-K12-11 apparently regulate RTA indirectly by targeting cellular genes that positively regulate RTA. A number of cellular transcription factors have been identified to activate the RTA promoter including MYB, Ets-1, and C/EBPα [28,29,30]. Lacoste and colleagues demonstrated that MYB transactivates the RTA promoter in the absence of any KSHV protein expression and furthermore that v-FLIP and v-GPCR induction of NF-κB leads to downregulation of MYB expression in PEL cells [29]. Our data indicate that miR-K12-11 directly targets the MYB 3'UTR (Figure 3) and that knockdown of miR-K12-11 increases MYB transcript level (Figure 4). Thus, miR-K12-11 fine tunes MYB expression post-transcriptionally in conjunction with virally encoded proteins that regulate MYB through NF-κB signaling. Interestingly, the NF-κB pathway or more specifically the IκBα super repressor, was shown to be regulated by miR-K12-1, thereby also inhibiting lytic growth [35].

Wang and colleagues observed that C/EBPα activates RTA directly and mapped three C/EBPα binding sites within the RTA promoter [30]. In addition, C/EBPα functions during lytic reactivation by interacting with RTA to bind and activate the K8 promoter, an early replication-associated protein. K8 then interacts with and stabilizes C/EBPα leading to upregulation of C/EBPα, in turn leading to further activation of RTA and K8 gene expression. It was also shown that C/EBPα activates additional early lytic genes PAN and ORF57 [30]. Even though miR-K12-3 does not appear to target C/EBPα directly by itself in 293 cells (Figure 3E), antagomir knockdown of miR-K12-3 in BCBL-1 cells does increase C/EBPα mRNA (Figure 4). Therefore, miR-K12-3 contributing to downregulation of C/EBPα represents targeting a central regulatory node to negatively modulate several immediate early and early genes.

Ets-1 was originally identified as an activator of RTA by utilizing BC-3-G cells in a genome-wide screen to identify cellular proteins and pathways that reactivate KSHV [28]. The Raf/MEK/ERK pathway was demonstrated to mediate KSHV reactivation and Ets-1, which is a downstream effector of this pathway, directly activates the RTA promoter. Ets-1 contains five putative KSHV miRNA binding sites in addition to a total of six seed sequence matches for miR-K12-3 and miR-K12-11. KSHV miRNA targeting of Ets-1 presumably contributes to maintenance of latency by inhibiting Raf/MEK/ERK induced activation of RTA. Ets-1 targeting by miRK-12-3 and -11 might be cell type specific since KSHV v-FLIP upregulates Ets-1 in a NF-κB-dependent fashion and moreover this is required for KSHV-dependent induction of lymphatic reprogramming of endothelial cells [58]. Hence, fine tuning of Ets-1 may be important to balance differentiation and reactivation. We recently demonstrated Ets-1 targeting in TIVE cells engineered to express miR-K12-11 in the absence of KSHV [59].

A common feature of these three transcription factors is their involvement in signaling pathways as part of cellular stress responses. The RTA promoter of γ-herpesviruses contains an array of transcription factor binding sites thereby sensing environmental changes and linking cell stress including innate and adapted immune responses against other pathogens to lytic reactivation [60,61]. Our data suggest a model where viral miRNAs contribute to the regulation of the latent to lytic transition by post‑transcriptionally modulating multiple signaling pathways. We believe that KSHV miRNAs fine‑tune the regulation of MYB, C/EBPα, and Ets-1, which increases the signaling threshold that has to be overcome before the lytic cascade can be initiated. Some of these cellular targets are regulated by other latency-associated genes, as in the case of MYB and NF-κB in lymphoid cells [29] and Ets-1 and NF-κB in endothelial cells [58,59]. Rather than acting like an on/off switch, viral miRNAs serve as gatekeepers of latency by manipulating multiple host cellular pathways that when activated would otherwise lead to RTA expression. In contrast to directly regulating RTA, targeting multiple cellular genes allows for greater flexibility in regulation of latency in different tissues and cell types where both the viral miRNAs and their cognate targets are expressed differentially [14,15,32,62]. For example, Ets‑1 levels are higher in endothelial cells while MYB is a master regulator of hematopoietic cells.

We propose that the regulation of host transcription factors by KSHV miRNAs contributes to the ability to quickly respond to environmental stimuli, which can overcome the intricate balance between miRNA copy number and their cognate targets [63,64]. A similar miRNA-based “spring-loaded” regulatory model was first proposed for OCT and SOX transcription factors that regulate pluripotency during differentiation [65]. In conclusion, our results suggest that miR-K12-3 and miR-K12-11 contribute to regulation of latency in cells of endothelial and lymphoid origin by targeting the host cellular transcription factors MYB, C/EBPα and Ets-1, thereby indirectly manipulating RTA expression.
